# Laterality influence on gene expression of DNA damage repair in colorectal cancer

**DOI:** 10.1038/s41598-023-42890-9

**Published:** 2023-09-25

**Authors:** Juliano Oliveira-Silveira, Eduardo Filippi-Chiela, Jenifer Saffi

**Affiliations:** 1 Centro de Biotecnologia, PPGBCM, Universidade Federal do Rio Grande do Sul, Porto Alegre, Rio Grande do Sul Brazil; 2 Departamento de Ciências Morfológicas, Instituto de Ciências Básicas da Saúde, Universidade Federal do Rio Grande do Sul, Porto Alegre, Rio Grande do Sul Brazil; 3 Departamento de Ciências Básicas da Saúde, Laboratório de Genética Toxicológica, Universidade Federal de Ciências da Saúde de Porto Alegre (UFCSPA), Porto Alegre, Rio Grande do Sul Brazil

**Keywords:** Tumour biomarkers, DNA damage and repair

## Abstract

Colorectal carcinoma (CRC) is the third most common malignancy worldwide, and second in number of deaths in the world. The molecular pathogenesis of CRC is heterogeneous and can affect several genes. Moreover, genomic instability is recognized as an important part of CRC carcinogenesis and is tightly connected to DNA damage response. DNA damage repair (DDR) pathways are intrinsically associated with cancer development and establishment. Traditionally, CRC is considered as one coherent disease, however, new evidence shows that left and right-sided CRC present differences observed in clinical settings, as well as in pre-clinical studies. Therefore, this study aimed to investigate the impact of DDR transcriptional profiles on survival in different sublocations of the colon and rectum using Cox regression, survival analysis and differential gene expression. Right side colon (RSC) has DDR genes’ expression associated only with higher risk of death, while left side colon (LSC) and Rectum have most genes’ expression associated with lower risk. The pattern is the same with survival analysis. All significant DDR genes had lower expression associated with better survival in RSC, as opposed to LSC and Rectum. Our results demonstrate that RSC is distinctively different from LSC and Rectum. LSC and Rectum have similar DDR expression profiles.

## Introduction

Cancers of the colon and rectum are the third most common malignancy worldwide, and over 1 million new cases are diagnosed each year. Colorectal carcinoma (CRC) is the third with the most cases and second in number of deaths in the world^1^. Both familial and sporadic cancers share a range of mutations and other molecular features^2^. The molecular pathogenesis of CRC is heterogeneous and can affect several genes, following a mutagenic succession, translated into a morphological sequence, starting with the formation of the adenoma, and ending in the carcinoma^3^.

The main drivers of CRC are mutations in *BRAF*, *NRAS*, and *KRAS* genes and Microsatellite Instability (MSI), while Consensus Molecular Subtypes (CMS)^4^ represent genomic markers for new classifications of CRC subtypes. Moreover, genomic instability is recognized as an important part of CRC carcinogenesis and is tightly connected to DNA damage response. DNA damage repair (DDR) pathways are intrinsically associated with cancer development and establishment for their dysregulation generates elevated mutation rates, increased genomic instability, and enhanced intra-tumor heterogeneity^5,6^. In CRC, DDR plays an important role in both carcinogenesis and response to therapy^7,8^.

CRC is traditionally treated as one cohesive disease, especially in pan-cancer studies^9^. More recent works separate colon and rectum, for their accentuated differences in short-term survival, complications, recurrence, and response to treatments^10^. In embryo development, the midgut gives rise to the distal duodenum, jejunum, ileum, cecum, ascending colon, and the proximal two-thirds of the transverse colon (right side). On the other hand, the hindgut develops into the distal one-third of the transverse colon, descending colon, sigmoid colon, and the rectum (left side and rectum)^11^. New evidence shows that left and right-sided CRC present differences observed in clinical settings, as well as in pre-clinical studies, including metastasis risk^12,13^, response to therapy^14^, immunogenicity^15^, histology^16^, sex- and age-related incidences^17^, and tumor microenvironment immune composition^18^. As can be seen, the main differences described so far concern the clinical behavior or histopathological characteristics of the tumors. Furthermore, the literature diverges on the impact of laterality on gene expression, drug response, and survival of CRC patients. However, the transcriptional landscape of DDR pathways and their clinical implications of prognosis had not yet been explored with laterality.

Therefore, in the present study, we aimed to investigate the impact of DDR transcriptional profiles on survival in different sublocations of the colon and rectum. Using a list of 189 genes of DDR pathways, we found that right side colon (RSC) is distinctively different from left side (LSC) and rectum. The left side and Rectum have similar expression profiles and share more molecular similarities.

## Materials and methods

### Data acquisition

We queried seven functional signaling pathways involved in DNA damage repair (DDR): homologous recombination (HRR), mismatch repair (MMR), base excision repair (BER), nucleotide excision repair (NER), nonhomologous end-joining (NHEJ), Fanconi anemia (FA), and translesion synthesis (TLS). The search resulted in 189 DDR genes which were used for further analysis. TCGA data were obtained from Broad GDAC Firehose (https://gdac.broadinstitute.org/, accessed on August 28th, 2020). Level 3 expression data for Colon and Rectum Adenocarcinoma (COADREAD) and available phenotype datasets were used. Primary tumor samples were analyzed, excluding metastatic tumor samples. Samples with unavailable relevant patient clinical information were also excluded.

### Cox regression

Multivariable Cox hazard regressions were performed for expression levels of queried genes shown in Suppl. Table 1 and adjusted for clinical variables according to the available information. Diagnostic analyses of the models were performed based on Martingale and weighted Schoenfeld residual plots and tests; clinical variables available in the baseline models were stratified to meet the proportional hazards assumption when necessary; patients were censored at 1800 days. Results were considered significant when the *p*-value < 0.05 (95% CI). Details of the models are provided in Suppl. Table 2.

### Survival analysis

For survival analysis, we used the Kaplan–Meier method to evaluate the correlation between overall survival time and gene expression. The median was used as a cutoff value for the classification of patients into high and low-expression groups. The curves were created after gene expression was discretized to the two groups. The statistical significance of overall survival was assessed using the Log-Rank test. All analyses were computed in the R statistical environment. Multivariable Cox proportional hazards, log-rank tests, and Kaplan–Meier curves were obtained using survival, ggplot2 and survminer packages in R (version 3.3.1) environment.

### Differential expression analysis

The differential gene expression analysis was performed on averaged CRC samples from each location to evaluate if they presented a statistically different value. The Wilcoxon rank test for paired data was applied to assess whether a gene was differentially expressed by comparing one sublocation with another. Statistical significance was determined using *p* < 0.05.

### Contingency tables and chi-squared

The CMS and MSI dataset were formatted as a contingency table using the gplots package, and graphical displays were generated using functions such as balloonplot and mosaicplot from the same package in R. The chi-square test of independence was performed using the chisq.test function, and the resulting Pearson residuals were extracted and visualized using the corrplot package.

## Results

### Colorectal cancer is not a cohesive disease

**Figure 1:**
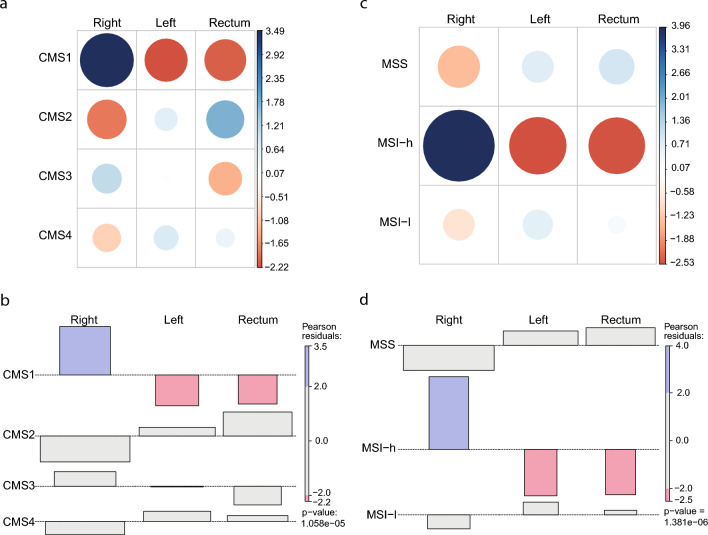
Consensus Molecular Subtype and Microsatellite Instability profiles are different in RSC from LSC and Rectum. (**a** and **c**): contingency tables for CMS and MSI status consecutively (*p* < 0.05). (**b**and **d**): Chi-square calculation with Peason residuals for CMS and MSI status consecutively. Pearson residuals were calculated to assess the nature of the dependence. Blue color indicates that the observed value is higher than the expected value if the data were random. Red color specifies that the observed value is lower than the expected value if the data were random. CMS1, Consensus Molecular Subtype 1; CMS2, Consensus Molecular Subtype 2; CMS3, Consensus Molecular Subtype 3; CMS4, Consensus Molecular Subtype 4. RSC, Right Sight Colon; LSC, Left Side Colon; MSS, Microsatellite stable; MSI-h, Microsatellite Instable High; MSI-l, Microsatellite Instable Low.

Initially, we investigated molecular aspects of colorectal cancer dividing the colon in left and right and comparing both with neoplasias of rectal origin. The Chi-square test demonstrates that CMS1 and high Microsatellite Instability are associated with RSC, suggesting that hallmarks of multiomic subtypes discretize the Colon on the Left and Right with distinct patterns (Fig. 1). LSC and Rectum, on the other hand, are not associated with any variable. Driver mutations present another marker of difference in RSC. When retrieving mutations from the browser COSMIC^19^, the top 13 mutated genes in RSC differ from left and rectum tumors, including BRAF high frequency in RSC, which characterizes CMS1 CRC subtype (Supp. Figure 1).

**Figure 2:**
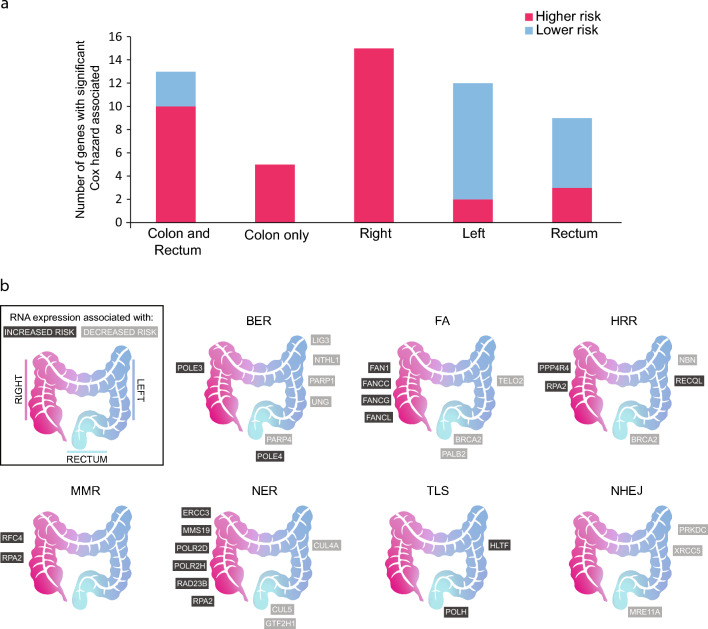
Cox hazard regressions using 189 DNA damage repair genes showing an overall protective state associated with the expression of DDR genes in the left side and a pattern of increased risk of death associated with expression in the right side. (**a**) Number of DDR genes with expression significantly associated with risk of death (Cox hazard, *p*-value < 0.05). (**b**) Scheme representing the laterality of gene expression of DDR. Genes with expression associated with increased risk of death (dark grey) or decreased risk of death (light grey). Base excision repair (BER), fanconi anemia (FA), homologous recombination (HRR), mismatch repair (MMR), nucleotide excision repair (NER), Nonhomologous End-Joining (NHEJ), and translesion synthesis (TLS).

To test the effect of segmentation of COADREAD dataset into Colon-Rectum, Left Colon-Right Colon and Rectum in risk of death association with expression of DDR genes, we performed Cox hazard regressions (*p*-value < 0.05) using 189 genes in each subgroup (Supp. Table S2, Supp File S4). Interestingly, there was a plethora of significant genes hidden in confounding factors when all samples were considered one group Colon and Rectum (13 genes). New genes showed up when the Colon only (5 genes) and Rectum (9 genes) were divided. Besides, more genes were discovered when Colon was further divided into Right (15 genes) and Left (12 genes) (Fig. 2a). When divided into three sections (Right, Left and Rectum) a total of 36 genes were associated with the risk of death.

### Subdivisions of colon and rectum unravel new biomarkers

Right side colon (RSC) is distinctively different from LSC and rectum. Cox regression analysis shows different effects of DNA damage repair (DDR) in different loci in the large intestine. Segmentation of CRC in the Colon and Rectum, and further colon in left and right, provide insight into the different profiles. When analyzed together (Colon and Rectum), only a fraction of the genes’ expression is significantly associated with decreased risk of death (Fig. 2b) (Supp. Table 2). When separated in the colon and rectum, the proportion of genes associated with decreased risk of death is augmented in the rectum, whereas in the colon subdivision the totality of genes is associated with increased risk of death. In further analysis, when we divided once again colon samples into RSC and LSC, we observed an even higher proportion of genes associated with a decreased risk of death in LSC, demonstrating a similar profile to that of rectum-located tumors.

### Left side colon is more similar to rectum than to right side

Among the DDR pathways, base excision repair (BER), fanconi anemia (FA), and nucleotide excision repair (NER) have the most genes with expression significantly associated with increased or decreased risk of death. NER and FA genes are predominantly associated with a higher risk of RSC while BER genes are mostly associated with decreased risk on LSC (Fig. 2b). Moreover, when analyzing BER, FA, HRR, and NER, it is possible to observe that LSC shares a protective state pattern with Rectal cancer, suggesting similarities between the two. In the mentioned pathways, most genes’ expressions are associated with decreased risk of death.

### Kaplan–Meier survival estimation curves also show the same opposing pattern

**Figure 3:**
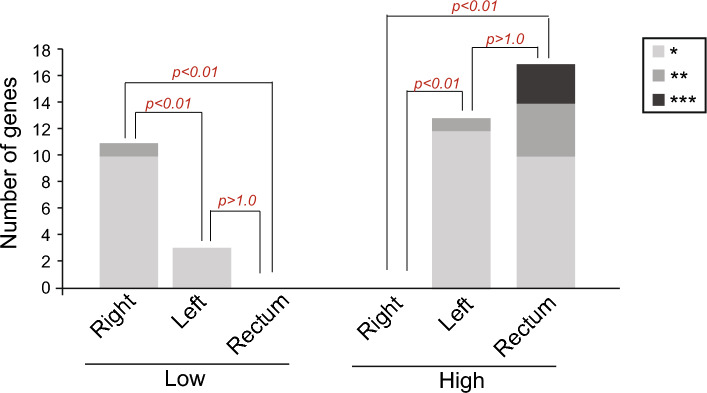
Summary of Kaplan–Meier survival estimation results. Number of DDR genes associated with increased survival and laterality. Median overall survival differences between high and low groups were assessed using the log-rank test. Patients were discretized by the median as low (lower than the median) or high (higher than the median). *p < 0.05; **p < 0.01; ***p < 0.001.

Kaplan–Meier curves were created after gene expression was discretized to “high” and “low” groups based on the median expression distribution of each queried gene (Supp. Files S1, S2, S3). Survival differences between both groups assessed using a log-rank test showed the same pattern with high expression of DDR genes increasing survival in samples from Left origin, while low expression increases survival in Right origin samples (Fig. 3). A transition pattern from low expression associated with better survival in RSC to high expression in the LSC illustrates the distinctiveness of RSC.

### Differential gene expression corroborates right/left-rectum divise

**Figure 4:**
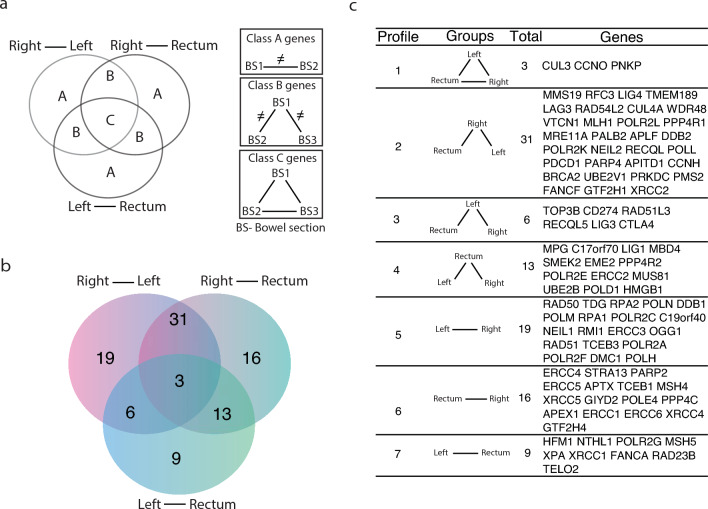
Comparison of differentially expressed genes. (**a**) Representative scheme guiding Venn interpretation (left) and gene expression comparisons between bowel section (BS) (Right). (**b**) Venn diagrams show the number of common and unique DEGs between different BSss. (**c**) Differentially expressed genes in each BS combination. Genes were classified in 7 profiles according to each combination. DEG analyses shown have *p*-value < 0.05.

Gene expression analysis demonstrates more evidence that LSC and Rectum share similarities and differ from RSC. In total 70% (66/97) of genes show differences between RSC and LSC or Rectum (Profiles 2, 5 and 6), with 32% (31/97) showing differences between RSC and LSC and Rectum at the same time (Profile 2) (Fig. 4). Out of the 31 Profile 2 genes, a sum of 22 DDR genes is underexpressed in RSC while a total of 10 is overexpressed. In the samples shown, genes BRCA2, PALB2, and MMS19 have indistinguishable expression levels in the rectum and left-sided samples, while both are distinguishable from RSC samples (Fig. 4).

## Discussion

In the last two decades, it has become a consensus that cancers differ from each other molecularly, even considering tumors originating from the same anatomical sites. In parallel to the characterization of intertumoral heterogeneity, omics studies have demonstrated the presence of physiologically distinct sub compartments in human organs. Among these organs is the large intestine, which presents distinct characteristics, for example, in the pattern of gene expression, microbiota composition, immune functioning, and inflammatory profile. Thus, it is plausible to infer that, from such different backgrounds, colorectal neoplasms present particularities associated with laterality. However, the molecular characterization of these differences remains poorly understood, especially considering key pathways in CRC carcinogenesis and outcome. Here, we characterized the expression profile of DDR pathways and its impact on the risk of death and prognosis of primary CRCs patients stratified by tumor location. Our work goes further to demonstrate the association of DDR genes with clinical parameters that would not be noted in the classical stratification of colon and rectal locations.

Here, we intended to show the transcriptional differences between CRC tumors originating from different sublocations, focusing on major DNA repair (DDR) pathways. Previous studies have shown right-side colon (RSC) tumors have a higher incidence of mutations in the DNA mismatch repair pathway and do not respond well to conventional chemotherapy^20^. To our thought, ours is the first study assessing the expression profile of main DDR pathways and its impact on the risk of death and prognosis of primary CRCs patients stratified by tumor location. Among the DDR pathways, base excision repair (BER), fanconi anemia (FA), and nucleotide excision repair (NER) have the most genes with expression significantly associated with increased or decreased risk of death. NER and FA genes have expression predominantly associated with higher risk on RSC while BER genes are mostly associated with decreased risk on LSC. Moreover, when analyzing BER, FA, HRR, and NER, it is possible to observe that LSC shares a protective state pattern with Rectal cancer, suggesting similarities between the two. In the mentioned pathways, most genes’ expressions are associated with decreased risk of death.

Therefore, the literature has brought relevant evidence considering the impact of laterality on the prognosis and risk of death in CRC. We found that patients from the LSC and rectum subsets had more DNA repair genes with expression associated with better survival. The right side, on the other hand, showed most genes with expression associated with worst survival. Here, we also assessed the risk of death for CRC patients considering DDR pathways. Compared to the entire cohort (i.e. RSC and LSC together), Cox regression showed different profiles of risk of death when CRC patients were divided into Colon and Rectum, and further divided into RSC, LSC, and Rectum. More significant genes are associated with risk when CRC is divided into three subsections (Right, Left and Rectum). Cox regression analysis showed that all significant DDR gene expressions in RSC were associated with an increased risk of death. Whereas, LSC showed more genes with expression associated with decreased risk of death. Petrelli et al. in a systematic review and meta-analysis analyzed 66 studies, with over 1.4 million subjects, and concluded that CRC patients with left-side origin have a 19% reduced risk of death when compared with right-side^21^. In this case, laterality was shown to have prognostic value independently of the stage, race, and adjuvant chemotherapy.

In general, considering the prognosis, there is a consensus that patients with right-side tumors have worse prognosis^22,23^. However, sidedness also influences the incidence and the impact of signaling pathways in the survival of CRC patients^24^, while this effect seems to be dependent on the genetic status of main driver genes, like KRAS and TP53. A meta-analysis identified a worse prognosis for RSC patients in KRAS wild-type tumors but not in KRAs-mutated tumors, at least in CRC patients with liver metastasis^25^. Furthermore, sidedness also impacts the survival of patients with metastatic tumors, since patients with recurrent liver metastasis from right-side tumors had shorter overall survival^26^. In a pioneer study, Sugai et al.^24^ observed an increased incidence of TP53 mutations in left-side CRCs. Posteriorly, Pan et al. assessed the influence of sidedness in the prognosis of CRC patients considering the status of TP53. The authors found that the poorer survival of patients with RSC in relation to LSC seems to be restricted to the subgroup of patients with non-gain-of-function TP53 mutation. Complementary to that, only LSC patients had poorer survival when comparing gain-of-function TP53 mutated patients versus those with no-gain-of-function TP53 mutation^27^. In Huang et al.^28^, the mutation landscape of CRC was explored, highlighting frequently mutated genes and their association with RSC and LSC. The study identified differences in mutation frequencies, with APC and TP53 mutations more common in left-sided CRC and PIK3CA mutations more common in right-sided CRC. The presence of DNA damage response (DDR) mutations was observed in both MSI and MSS CRC.

Finally, we also investigated differences in CMS and MSI, which are clinically relevant variables in CRC. Our results corroborate previous studies showing increased incidence of CMS1 and MSI among right-sided tumors^4,29^. The MSI profile impacts, for instance, lymphocyte infiltration^30^ and response to therapy^31^, so the differences in MSI observed here could underlie the differences in the immune composition of tumor microenvironment^18^ and in the sensitivity to classic chemotherapy and targeted therapies^32,33^ between right and left CRC tumors. For example, patients with KRAS/RAS-wt mCRC treated with first-line EGFR-I plus chemotherapy with a LSC primary tumor showed improved results in a drug trial when compared to RSC patients^14^. Furthermore, the specific differences in tumor microenvironment between right and left colorectal tumors may also contribute to explaining the differential impact of immune-associated markers in the prognosis and response to immunotherapy. For instance, PD-L1 expression is higher in right than left-side tumors and associated with worse prognosis in left-side cancer. The same association was observed with the Treg-specific marker Foxp3^18^.

## Conclusion

These results together suggest that DDR gene expression effect on Right Side Colon is distinct from the other two subdivisions, and Left Side Colon and Rectum have similar profiles and likely share more molecular similarities, including those associated with response to DNA damaging agents such as chemotherapy. Because DNA damage response is an important aspect of tumor response to different therapies, tumor localization should be accounted for when developing and testing new drugs or electing subjects for clinical trials. Treating all samples as one cohesive disease may lead to confounding effects that hide potential therapeutic benefits in subgroups, which would otherwise be mistaken for null results. Furthermore, DDR gene expression levels should not function as risk of death and prognostic markers for patients with CRC unless the anatomical site of the tumor is taken into account. In conclusion, right side primary tumors should be treated as a different neoplasm from tumors with a left/rectum origin due to its DDR gene expression landscape.

### Supplementary Information

Supplementary Tables.

## Data Availability

The current research and analysis data sets come from the online database the Cancer Genome Atlas (TCGA, http://can-cergenome.nih.gov/). Processed data and R language code used in the analysis are available in the Supplemental Material file (Supp. Files S5, S6, S7, S8). Other data supporting the results of this study can be obtained from the corresponding author upon reasonable request.
