# Supplementary material for: Laterality influence on gene expression of DNA damage repair in colorectal cancer
Source: Sci Rep. 2023 Sep 25;13:15963. doi: 10.1038/s41598-023-42890-9 (PMC10519976; doi:10.1038/s41598-023-42890-9)
Supplement: Supplementary file 1 — Supplementary Tables. [file 41598_2023_42890_MOESM1_ESM.docx]

Supplementary Table S1. **Queried genes for survival and differential expression analyses.** Homologous recombination (HRR), mismatch repair (MMR), base excision repair (BER), nucleotide excision repair (NER), nonhomologous end-joining (NHEJ), Fanconi anemia (FA), and translesion synthesis (TLS).

| Pathway | Genes |
| --- | --- |
|  |  |
| BER (n = 31) | APEX1, APLF, APTX, CCNO, FEN1, HMGB1, LIG1, LIG3, MBD4, MPG, MUTYH, NEIL1, NEIL2, NEIL3, NTHL1, OGG1, PARP1, PARP2, PARP3, PARP4, PCNA, PNKP, POLB, POLD1, POLD2, POLD3, POLD4, POLE, POLE2, POLE3, POLE4, POLL, SMUG1, TDG, TDP1, UNG, XRCC1 |
|  |  |
|  |  |
| FA (n = 25) | CENPS, BLM, BRCA1, BRCA2, BRIP1, FAAP100, FAAP24, FAN1, FANCA, FANCC, FANCD2, FANCE, FANCF, FANCG, FANCI, FANCL, FANCM, HES1, PALB2, RAD51, RAD51C, RMI1, RMI2, CENPX, TELO2, TOP3A, TOP3B, UBE2T, USP1, WDR48 |
|  |  |
| HRR (n = 38) | BLM, BRCA1, BRCA2, DMC1, EME1, EME2, GEN1, HFM1, MRE11, MUS81, NBN, PPP4C, PPP4R1, PPP4R2, PPP4R4, RAD50, RAD51, RAD51B, RAD51C, RAD51D, RAD52, RAD54B, RAD54L, RAD54L2, RDM1, RECQL, RECQL4, RECQL5, RMI1, RMI2, RPA1, RPA2, RPA3, SEM1, SLX1A, SLX4, PPP4R3A, PPP4R3B, SPO11, TOP3A, TOP3B, WRN, XRCC2, XRCC3 |
|  |  |
| MMR (n = 25) | EXO1, HMGB1, LIG1, MLH1, MLH3, MSH2, MSH3, MSH4, MSH5, MSH6, PCNA, PMS1, PMS2, POLD1, POLD2, POLD3, POLD4, RFC1, RFC2, RFC3, RFC4, RFC5, RPA1, RPA2, RPA3 |
|  |  |
| NER (n = 42) | BIVM-ERCC5, CCNH, CDK7, CUL3, CUL4A, CUL5, DDB1, DDB2, ERCC1, ERCC2, ERCC3, ERCC4, ERCC6, ERCC8, GTF2H1, GTF2H3, GTF2H4, GTF2H5, LIG1, MMS19, MNAT1, POLR2A, POLR2B, POLR2C, POLR2D, POLR2E, POLR2F, POLR2G, POLR2H, POLR2I, POLR2J, POLR2J2, POLR2K, POLR2L, RAD23A, RAD23B, RBX1, RPA1, RPA2, RPA3, ELOC, ELOB, ELOA, ELOA2, ELOA3B, XPA, XPC |
|  |  |
| NHEJ (n = 15) | APLF, APTX, DCLRE1C, DNTT, LIG4, MRE11A, NHEJ1, POLB, POLL, POLM, PRKDC, RAD50, XRCC4, XRCC5, XRCC6 |
|  |  |
| TLS (n = 13) | HLTF, POLH, POLI, POLK, POLN, RAD18, REV1, REV3L, TMEM189, UBE2B, UBE2N, UBE2V1, UBE2V2 |
|  |  |

Supplementary Table S2. **Risk values for DDR genes in each sublocation.** Negative risk values represent negative association between risk and gene expression. Positive risk values represent positive association between risk and gene expression. Results shown with p < 0.05.

|  |  | COLON | | | RECTUM | COLON & RECTUM |
| --- | --- | --- | --- | --- | --- | --- |
|  | Gene | Full | Right | Left | Full | Full |
| BER | LIG3 | - | - | -4.28 | - | - |
|  | NTHL1 | - | - | -4.37 | - | - |
|  | PARP1 | - | - | -8.37 | - | - |
|  | PARP2 | - | - | - | - | 1.91 |
|  | PARP4 | - | - | - | -3.39 | - |
|  | POLE3 | - | 2.49 | - | - | - |
|  | POLE4 | - | - | - | 3.14 | - |
|  | UNG | - | - | -3.98 | - | - |
| MMR | MSH4 | - | - | - | - | 1.24 |
|  | RFC4 | - | 1.95 | - | - | - |
|  | RPA2 | - | 2.15 | - | - | 1.82 |
| FA | CENPX |  |  | -4.34 | - | - |
|  | FAN1 |  | 2.20 | - | - | - |
|  | FANCC | - | 2.01 | - | - | - |
|  | FANCG | - | 2.44 | - | - | - |
|  | FANCL | - | 2.23 | - | - | - |
|  | TELO2 | - | - | -5.80 | - | - |
|  | BRCA2 | - | - | - | -2.31 | - |
|  | WDR48 | - | - | - | - | -2.18 |
|  | PALB2 | - | - | - | -7.47 | - |
| HRR | BRCA2 | - | - | - | -2.31 | - |
|  | NBN | - | - | -5.14 | - | -1.62 |
|  | PPP4R4 | 1.28 | 1.47 | - | - | - |
|  | PPP4R2 | - | - | - | - | 1.73 |
|  | RECQL | - | - | 9.04 | - | - |
|  | RPA2 | - | 2.15 | - | - | 1.82 |
|  | XRCC2 | 1.63 | - | - | - | - |
| NER | CUL4A | - | - | -12.59 | - | - |
|  | CUL5 | - | - | - | -3.84 | - |
|  | ERCC3 | - | 4.64 | - | - | - |
|  | GTF2H1 | - | - | - | -5.65 | - |
|  | MMS19 | 2.05 | 3.75 | - | - | 1.90 |
|  | POLR2D | - | 3.68 | - | - | 1.73 |
|  | POLR2H | - | 2.19 | - | - | - |
|  | RAD23B | - | 3.69 | - | - | - |
|  | XPC | - | - | - | - | -2.18 |
|  | RPA2 | - | 2.15 | - | - | 1.82 |
| NHEJ | PRKDC | - | - | -3.14 | - | - |
|  | XRCC5 | - | - | -13.61 | - | - |
|  | DCLRE1C | - | - | - | - | 1.66 |
|  | MRE11A | - | - | - | 5.74 | - |
| TLS | HLTF | 1.42 | - | 2.06 | - | - |
|  | TMEM189 | 2.16 | - | - | - | - |
|  | POLH | - | - | - | 4.11 | - |
|  | TMEM189 | - | - | - | - | 2.03 |
